# Astrocytic estrogen receptors and impaired neurotrophic responses in a rat model of perimenopause

**DOI:** 10.3389/fnagi.2015.00179

**Published:** 2015-09-29

**Authors:** Todd E. Morgan, Caleb E. Finch

**Affiliations:** Davis School of Gerontology, Department of Biological Sciences, Dornsife College, University of Southern CaliforniaLos Angeles, CA, USA

**Keywords:** estrogen receptors, perimenopause, astrocytes, glial fibrillary acid protein, neurotrophic

## Abstract

In a perimenopausal model of middle-aged rats, the astrocyte estrogen receptor-alpha (ERa): ER-beta (ERb) ratio increased with the onset of acyclicity (constant estrus, CE) in association with impaired neurotrophic responses to estradiol (E2). We report additional data on irregular cycling (IR) from this study of 9 month old perimenopausal subgroups. In particular, irregular cyclers (IR) also show increased ERa:ERb ratio in cerebral cortex astrocytes comparable to acyclic individuals in CE. In mixed glial cultures from these same cycling subgroups, the E2-dependent neurotrophic activity and glial fibrillary acidic protein (GFAP) repression by E2 were impaired in IR to the same degree as in CE-derived glia. The greater importance of cycling status than age during the perimenopause to astrocyte ERs are attributable to individual variations of the residual ovarian follicle pool, which determine the onset of acyclicity. The corresponding loss of E2-dependent GFAP repression and E2-dependent neurotrophic activity add further to the inverse relationship of GFAP expression and astrocyte neurotrophic activity across aging in both sexes. These findings are relevant to impairments of spatial learning and of hippocampal long-term potentiation during the onset of IR in middle-aged rats, and to perimenopausal factors mediating the higher risk of women for Alzheimer disease.

## Introduction

Estrogen receptor-alpha (ERa) and estrogen receptor-beta (ERb) undergo age-related shifts in both sexes (Wilson et al., 2002; Wu et al., 2009; Arimoto et al., 2011, 2013; Arimoto, 2012; Foster, 2012). In brain, ER aging changes vary by cell type and hormonal status. In a perimenopausal model of middle-aged rats, the astrocyte ERa:ERb ratio was increased in acyclic constant estrus (CE) rats (Arimoto et al., 2013). The ERa:ERb ratio has broad significance to synaptic plasticity (Foster, 2012; Bean et al., 2014), which we have characterized for astrocytic neurotrophic support (Rozovsky et al., 2002, 2005; Arimoto et al., 2011, 2013). In particular, elevations of ERa:ERb decrease neurotrophic support, which is indirectly linked to expression of glial fibrillary acidic protein (GFAP), the astrocytic intermediate filament through its estrogen response elements (Rozovsky et al., 2002; Stone et al., 1998). The elevated ERa:ERb in astrocyte cultures from aging males was manipulated by RNAi, which decreased GFAP (Rozovsky et al., 2005; Arimoto et al., 2013). Both E2 responses (GFAP repression and induction of neurotrophic activity) are lost during aging (Rozovsky et al., 2005; Arimoto et al., 2013). In this review, we present data on irregular cycling (IR; Arimoto et al., 2011) that extends findings of Arimoto et al. (2013) and discuss how changes in astrocytic ERs during the perimenopause transition may result from changes in steroids from both the ovary and the brain. The importance of IR status was shown in impaired learning of IR rats vs. RC (regular cycling) of the same age (Paris et al., 2011) and impaired long-term potentiation in hippocampal slices (Yin et al., 2015).

A group of 9 month old rats were characterized for cycling status: regular cycles 4–5 day (RC); irregular cycles >5 day (IR); and acyclic CE. The IR group was not reported in Arimoto et al. (2013) but was described in Arimoto et al. (2011) and the Ph.D. Thesis of Jason Arimoto (USC Department of Biological Sciences, 2012). First we describe astrocyte ER *in vivo* expression in 9 month old rats. Then we describe primary glial cultures from the same groups for astrocyte GFAP expression in relation to neurotrophic activity.

## Astrocyte ERa:ERb Ratio Increases with Age

**Figure 1:**
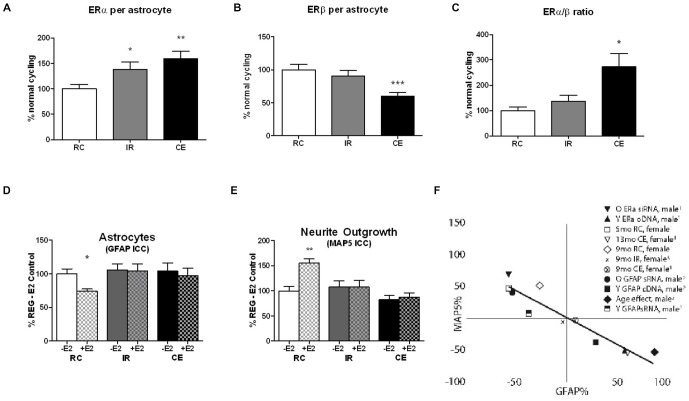
**(A–C)** *In vivo* ERα and ERβ expression in cerebral cortex astrocytes of perimenopausal female rats, aged 9 month differ by ovarian cycle status: regular cycles (RC) 4–5 day irregular cycles (IR) >5 day; constant estrus (CE) (Nelson et al., 1982; Finch, 2014). Astrocyte ERs in primary visual cortex in layers 1 and 2/3, RC vs. IR vs. CE; 4 brains per cycle status; Data is expressed as % regularly cycling (RC) control; mean+SEM; ***p < 0.001; **p < 0.01; *p < 0.05. RC and CE data published in Arimoto et al. (2013), Figure 7 (n.b. CE is denoted as AC, ibid.). The IR data are from Arimoto et al. (2011) and the thesis of Jason Arimoto (USC, 2012). **(D,E)** *In vitro* GFAP expression and neurotrophic activity (MAP5, neurite outgrowth) in mixed glia from 9 month old perimenopausal rats of different cycling status: RC vs. IR vs. CE. Mixed glial were co-cultured with E18 neurons; 4 cultures, 4 brains per cycling stage group. Data expressed as % RC control; mean + SEM; **p < 0.01; *p < 0.05. GFAP responses to 100 pM E2 in mixed glia from 9 month rats: The RC responded to E2 with decreased GFAP, but age-matched IR and CE were unresponsive. Neurite outgrowth (MAP5) was increased by 100 pM E2 in RC, but not in age-matched IR and CE. The RC and CE data were published in Arimoto et al. (2013), Figure 6 (n.b. CE is denoted as AC, ibid.). The IR data are from Arimoto et al. (2011) and the thesis of Jason Arimoto (USC, 2012). **(F)** Reciprocal relationships between GFAP and neurite outgrowth (MAP5), plotted as % change relative to controls in that experiment. RC and CE data published in Arimoto et al. (2013), Figure 8. New data on 9 month IR are calculated from **(D,E)**.

Age changes in astrocyte estrogen receptors (ER) are under-represented in the growing literature on ERs in brain aging, which has mainly focused on neuronal ERs (Bean et al., 2014; Kermath et al., 2014). Both sexes of aging rats (9–24 month, middle-age to old age) show increased ERa:ERb in astrocytes of cerebral cortex as an outcome of increased ERa and decreased ERb, as determined from immunohistochemical analysis of co-labeling for GFAP, the astrocyte-specific protein (Arimoto et al., 2013). Figure 1 shows progressive increase of ERa and decrease of ERb during transitions from RC to IR to CE (Figures 1A,B). In the prior comparison of CE with RC (Arimoto et al., 2013), ERb levels were lower by 40%, whereas IR were just 10% lower (Figure 1B). Correspondingly, the ERa:ERb ratio increased by 1.2-fold in IR and 2.5-fold in CE (Figure 1C). In an older cohort at 13 month, CE and IR had similar shifts of ERa:ERb (Arimoto et al., 2013, not shown). Primary glial cultures of enriched astrocytes or mixed glial (astrocytes: microglia, 3:1) retain these age changes. Perimenopausal rats age 9 month show further complexity in comparisons of regular cyclers (RC) vs. irregular cyclers (IR) vs. acyclic CE. The data on IR (Arimoto et al., 2011; Arimoto, 2012) show ERa:ERb shifts *in vivo* that are comparable to the CE in 9 month old rats (Figure 1A). We suggest that astrocytes may be major contributors to the decrease of ERb in whole hippocampal RNA from IR vs. RC rats (Yin et al., 2015).

## Inverse Relationships between GFAP and Neurotrophic Activity

Primary cultures of mixed glia (astrocytes:microglia, 3:1) were prepared from the other hemicortex from these rats (Arimoto et al., 2013). Mixed glia were used rather than enriched astrocytes because we wished to avoid artifactual perturbation of gene activity associated with hydrodynamic effects of shaking to remove microglia (Gatson et al., 2011). In male derived glia, mixed glia and enriched astrocytes showed the same age trends in ERs and GFAP, and neurotrophic activity (Arimoto et al., 2013). In glia from 9 month rats, astrocyte GFAP was repressed by E2 in RC, whereas IR and CE were unresponsive to E2 (Figure 1D). Correspondingly, neurotrophic activity in conditioned media assayed by neurite outgrowth was increased by E2 only in glia from regular cyclers (Figure 1E). The reciprocal relationship between GFAP expression and astrocyte neurotrophic activity (RC vs. IR vs. CE) is consistent with prior analysis, updated in Figure 1F.

Enhanced GFAP expression influences neurite outgrowth through astrocyte-secreted laminin and other extracellular factors (Rozovsky et al., 2002). Loss of E2-sensitivity of neurite outgrowth in male derived astrocytes was reversed by down regulation of GFAP by RNAi; conversely, young astrocytes acquired an aging phenotype by transfection with GFAP cDNA (Rozovsky et al., 2005). Because GFAP is regulated by E2 through a classical promoter ERE that binds ERa (Stone et al., 1998), we examined the role of ERs. The elevated ERa:ERb in astrocyte cultures from aging males was manipulated by RNAi to ERa, which decreased GFAP (Arimoto et al., 2013). However, neurotrophic activity was not restored, suggesting additional ER dependent mechanisms. The loss of E2 responses in GFAP repression and in E2-dependent neurotrophic activity may be the earliest impairment in gene responses in a rodent model.

## Perimenopausal Transition

**Figure 2:**
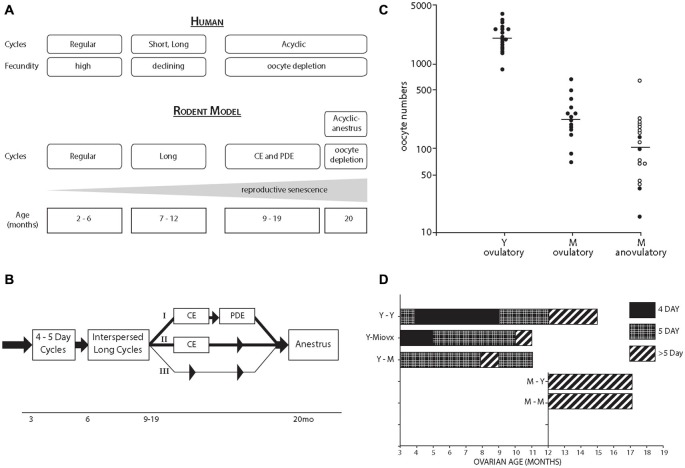
**(A)** Comparison of perimenopausal transitions in women and rodents (lab mouse and rat). The most common initial acyclic state of rodents, CE is driven by continued ovarian estradiol (E2) production at low levels with hypothalamic impairments of the E2-dependent preovulatory gonadotrophin surge. Alternate pathways with persistent diestrus (PDE) are shown in panel **B**. The terminal state of anestrus with total oocyte depletion arise after 20 month, with LH elevations as in postmenopausal women. Rodent perimenopause occupies a larger fraction of the 28 month life span than for women, spanning the onset of cycle irregularity after 6 month to ovarian follicle depletion after 20 month, which comprises half the lifespan. For details, see Nelson et al., 1982; Felicio et al., 1984; Finch et al., 1984; Gee et al., 1983; Finch, 2014. **(B)** Rodent perimenopausal transitions with alternate trajectories from lengthening cycles interspersed with 4–5 day ovulatory cycles (I) CE followed by PDF (repetitive pseudopregnancy), and thence to anestrus; (II) CE directly to anestrus; (III) Lengthening cycles leading directly to anestrus (least common). Adapted from Finch et al. (1984), **(C)** Studies on C57BL/6J mice from the Finch lab. Ovarian oocyte numbers in Y (young, 4–5 month) and M (middle-aged, 13–14 month); the M were grouped by the occurrence of recent ovulation. Horizontal lines are median values. From Gosden et al. (1983). **(D)** Heterochronic ovary transplantation between Y and M ages. The MLovx were ovariectomized (OVX) at 3 month and given ovaries at 12 month; their aging without exposure to ovarian steroids maintained the potential for 4 day cycling, not seen in Y-M transplant. Redrawn from Felicio et al. (1986).

The rodent perimenopausal model (Figures 2A,B) is described with reference to the STRAW stages of human menopause (Finch, 2014). Rodents and humans undergo similar ovarian senescence, beginning with increasingly IR and declining fertility, and ending with total depletion of ovarian follicles (Finch et al., 1984). The CE following IR with modest sustained blood E2 and very low progesterone (P4) may be considered as a model for hyperestrogenic cycles during human peri-menopause (Finch, 2014). We hypothesize that the perimenopause may be characterized by a general trend for increasing plasma E2:P4 during the perimenopause (Finch et al., 1984; Finch, 2014), equivalent to progressive exposure to unopposed estrogen.

To study perimenopausal changes associated with cycle lengthening, we used a mixed glia model (3:1 astrocytes; microglia), which had shown the same age changes of ERa, GFAP, and neurotrophic activity as enriched astrocytes in males (Arimoto et al., 2013). Mixed glia from irregularly cycling 9 month rats and CE rats both show impaired E2-responses of GFAP and of neurotrophic activity. Thus, the loss of hypothalamic GFAP responses to E2 in middle-aged rats (Anderson et al., 2002) has a counterpart in cerebral cortex astrocytes. Moreover, it shows the impact of modest steroidal perturbations associated with irregular cycles to synaptic functions related to memory. In IR vs. RC of the same age, hippocampal slices show impaired LTP (Yin et al., 2015), while intact rats show impaired spatial learning (Paris et al., 2011). In an astrocyte-neuron culture model, ERa was specifically associated with E2-induced glutamatergic synaptogenesis (Jelks et al., 2007). We suggest that the impairments of E2-dependent neurotrophic activity in IR rats can be used to identify E2-dependent neurotrophic factors underlying LTP and spatial memory.

## Ovarian Contributions

We suggest that the early onset of ER changes in females is driven by ovarian steroidal changes. In particular, as estrous cycles undergo lengthening and transition to acyclicity, there is a modest reduction of the blood estradiol: progesterone ratio (E2:P4) (Finch et al., 1984). At much later ages, typically after 20 month in rodents, ovarian follicles are depleted with reductions of plasma E2 to ovariectomized (OVX) levels (Gee et al., 1983). Although male rodents do not undergo total reproductive senescence with the complete loss of fertility and gonadal steroids in older age, plasma testosterone tends to decrease in middle-aged male rats (Rosario et al., 2009).

Further perspectives come from studies of 3 decades ago in our lab. By 12–14 month, rodent ovaries have < 10% of oocytes and primary follicles remaining from the young adult (Figure 2C; Gosden et al., 1983). Note the wide range of oocyte numbers; this 5-fold wide range is hypothesized to underlie the individual differences in onset of acyclicity (Finch and Kirkwood, 2000).

The role of oocyte depletion in cycle lengthening was shown with ovarian transplants between young and middle-aged mice (heterochronic transplants; Figure 2D; Felicio et al., 1986). Replacing young ovaries with middle-aged ovaries caused premature cycles lengthening to >5 days. Despite the wide range of remaining follicles in middle-aged ovaries (Figure 2C), the remaining number is close to the threshold required for ovulatory cycles. The tight linkage of cycle lengthening to the remaining ovarian follicles is also shown by the induction of premature cycle lengthening and CE by surgically removing 90% of young ovarian mass (Nelson and Felicio, 1986).

## Neuroendocrine Contributions

Neuroendocrine mechanisms are also at work, as shown by transplant of young ovaries to middle-aged mice, which yielded mostly 5 day cycles. However, if middle-aged mice were OVX when young and allowed to age without the presence of ovaries (long-term ovariectomy, Lt-OVX), then 4 day cycles were observed. Thus, the loss of 4 day cycles has a neuroendocrine component that is modified by estrogen exposure. The estrogen exposure hypothesis of cycle lengthening was tested by several durations of exposure to exogenous E2, which advanced the onset of acyclicity, in which the neuroendocrine component was shown by transplantation of control ovaries (Mobbs et al., 1985; Kohama et al., 1989b). As few as 6 weeks of low sustained E2 accelerated the onset of acyclicity (Kohama et al., 1989b). The importance of the E2:P4 ratio was shown by the protective effect of P4 implants (Kohama et al., 1989a). We attribute these effects of E2:P4 on synaptic remodeling rather than neurodegeneration because chronic E2 did not change the number of hypothalamic LHRH or TIDA neurons (Kohama et al., 1992). Astrocyte GFAP is a mediator of estrous cycles by the close relation of GFAP-containing astrocyte processes to the LHRH neurons which shift during proestrus (Garcia-Segura et al., 1994). The E2-dependent induction of hypothalamic GFAP was impaired in middle-aged rats in parallel with the loss of the E2-induced LH surge (Anderson et al., 2002). Subsequently, we developed *in vitro* systems for studying GFAP regulation that further documents impaired GFAP responsiveness with functional connections to neuronal plasticity.

## Conclusion

Laboratory rodents show a perimenopausal transition with lengthening cycles that has both brain (neuroendocrine) and ovarian contributions, as shown by prior ovarian transplantation studies between different age groups. Recent data further show that the neurotrophic activity of astrocytes assayed *in vitro* declines in the earliest perimenopausal stage of cycle irregularity and with cycle lengthening. These findings are relevant to impairments of spatial learning and of hippocampal long-term potentiation during the onset of IR and to perimenopausal factors mediating the higher risk of women for Alzheimer disease.

## Conflict of Interest Statement

The authors declare that the research was conducted in the absence of any commercial or financial relationships that could be construed as a potential conflict of interest.
